# Counting the cost of public and philanthropic R&D funding: the case of olaparib

**DOI:** 10.1186/s40545-022-00445-9

**Published:** 2022-08-16

**Authors:** L. Schmidt, O. Sehic, C. Wild

**Affiliations:** Austrian Institute for Health Technology Assessment GmbH, Garnisongasse 7/20, 1090 Vienna, Austria

**Keywords:** Research funding, Public funding, Return on investment, Pharmaceutical R&D

## Abstract

**Background:**

Lack of transparency around manufacturing costs, who bears the bulk of research and development costs and how total costs relate to the pricing of products, continue to fuel debates. This paper considers the case of olaparib (Lynparza®), recently indicated for use among BRCA-mutant breast cancer patients, and estimates the extent of public and philanthropic R&D funding.

**Methods:**

We know from previous work that attempting to ascertain the amount of public and philanthropic funding using purely bibliographic sources (i.e., authors’ declarations of funding sources and amounts traced through funders) is limited. Since we knew that a publically funded research unit was pivotal in developing olaparib, we decided to supplement bibliographic data with a Freedom of Information request for administrative records on research funding data from this research centre.

**Research:**

In terms of stages of product development, work conducted in the pre-clinical research stage was the most likely to report non-industry funding (> 90% of pre-clinical projects received public or philanthropic funding). Clinical trials were least likely to be funded through non-industry sources—although even here, contrary to the popular assertion that this is wholly industry-financed, we found public or philanthropic funding declared by 23% of clinical trials. Using information reported in the publications, we identified approximately £128 million of public and philanthropic funding that may have contributed to the development of olaparib. However, this amount was less than one-third of the total amount received by one research institute playing a pivotal role in product discovery. The Institute of Cancer Research reported receiving 38 funding awards to support olaparib work for BRCA-mutant breast cancer totalling over £400 million.

**Conclusions:**

Government or charitable funding of pharmaceutical product development is difficult to trace using publicly available sources, due to incomplete information provided by authors and/or a lack of consistency in funding information made available by funders. This study has shown that a Freedom of Information request, in countries where such requests are supported, can provide information to help build the picture of financial support. In the example of olaparib, the funding amounts directly reported considerably exceeded amounts that could be ascertained using publically available bibliographic sources.

## Background

The debate about whether high drug prices are justified by the cost of pharmaceutical development, given the considerable funding for research and development (R&D) that comes from the public sector and charities, continues to rage. This debate has been further fuelled by the current worldwide reliance on a few COVID-19 vaccines marketed by pharmaceutical companies and the inability of many poorer countries to secure access. A recent study has estimated the net costs for 100 million doses of COVID-19 vaccine ready for shipping to be very low; considerably lower than current market prices for vaccine doses [1]. The COVID-19 vaccines are an example of the lack of transparency and secrecy that often accompanies this debate: secrecy around manufacturing costs; a lack of transparency about development costs and who contributes to these; and negotiations and contracts between industry and government, which are not made public.

It has been said that the public pays twice for pharmaceuticals—once when basic research is funded by the public sector, and then again when high prices are paid by governments and individuals for the resulting products [2]. Our previous work has shown that it is not only basic research that is funded by the public; considerable charity, national government and supranational organisations (such as the European Union or EU) funding supports application-specific later stage development research too. Recent work by Nayak et al. [3], who investigated downstream public sector support by examining patent and drug development histories, revealed about two-fifths of new biologic drugs approved by the United States (US) Food and Drug Administration (FDA) between 2008 and 2017 had received financial support from public sector institutions or their spin-offs for late-stage development. This follows their earlier work, demonstrating that publicly supported research had a major role in the late stage development of one in four new drugs [4].

In terms of the actual quantification of the support public funding confers, estimates vary. This is likely to be as much down to the method used for estimating financial support, as variations between the products themselves. The Tufts Center for the Study of Drug Development has estimated that it costs pharmaceutical companies $2.6 billion to develop a new drug [5]. More than 80% of new compounds are estimated to be abandoned at some point in their development—a key driver of the Tufts cost estimate (made up of both the costs of developing successful products, but also the costs of those that never reach the market, i.e., risk capital). Others too have attempted to estimate the R&D costs associated with developing new drugs. A review of estimates available in the published literature shows a wide range in estimated development costs, from $43.4 million to $4.2 billion [6]. Behind these estimates lie a variety of methods and data sources, many of which are confidential, which makes comparison difficult.

In our previous work, we assessed the amount of governmental or charity funding that had gone into the later-stage development of three paediatric orphan drugs and generated a conservative estimate of between €20 and €31 million [7]. We also looked at the specific role of a large European Union funding programme (Seventh Framework Programme for Research of the European Union, known as ‘FP7’) in funding the development of an orphan drug and the development of a vaccine—the latter illustrating the role of public funding as risk capital [8]. In this paper, we apply bibliographic methods to a different scientific area (poly-ADP ribose polymerase or PARP inhibitors for breast cancer). Olaparib was chosen as it was developed by a public sector research institute in the United Kingdom (UK) and is marketed by a British pharmaceutical company. Here we complement the bibliographic approach, tested in our earlier work, with data on funding acquired from the research centre, which was pivotal in the product’s scientific development.

## Methods

We used three approaches to identify the scientific work, and its funding, that was crucial to the development of olaparib. First, we conducted bibliographic database (PubMed) searches to identify (i) publications relating to primary research undertaken in the development, or clinical testing, of olaparib and (ii) reviews describing the development of olaparib. Second, we identified scientific work cited in the patents relating to olaparib. Third, we made a Freedom of Information (FOI) request to the principal research organisation involved in product development (Institute of Cancer Research, London or ICR), asking for information on the funding of research into the development of olaparib. Each approach is described in detail in turn below.

We carried out two PubMed bibliographic database searches to identify relevant primary and secondary publications. The first was a search based on the names of the main discoverers that had previously been identified via a breast cancer charity news report describing the development of PARP inhibitors in the fight against breast cancer [9]. This search was carried out on August 8th 2021: *[(Ashworth OR King OR Lord OR Tutt) AND (PARP OR Lynparza OR Olaparib)]*. The second bibliographic database search related to the clinical trials landscape, carried out on December 5th 2021: *[(lynparza OR olaparib) AND breast Filters: Clinical Study, Clinical Trial, Clinical Trial, Phase I, Clinical Trial, Phase II, Clinical Trial, Phase III, Clinical Trial, Phase IV, Controlled Clinical Trial, Multicenter Study, Observational Study, Randomized Controlled Trial, Validation Study].*

We applied inclusion and exclusion criteria to the results of the bibliographic searches as follows. *Included* were: review articles describing the development of PARP inhibitors for breast cancer; preclinical research, e.g., describing animal models; proof of concept clinical trials or any trial phases I to III relating to olaparib for breast cancer treatment. *Excluded* were: clinical trials relating to combination therapies of olaparib with other therapies; studies published after the product received EU approval in 2019; experimental studies that did not appear part of the development pathway.

Review articles identified through the two PubMed searches were used to identify principally pre-clinical research that had been pivotal to the development pathway. The inclusion and exclusion criteria were similarly applied to any studies additionally identified through the review articles.

The second method used to identify relevant scientific work in the development of olaparib was through patents, although this was for indicative purposes only. One of the pre-clinical studies identified reported that KuDOS Pharmaceuticals Ltd and the ICR submitted a patent application based on their results [10]. The full text of publications cited in these patents were then retrieved. We have included scientific work referenced in patents as indicative only, due to issues in apportioning this type of basic research to specific applications. We did not apply the inclusion and exclusion criteria to scientific work cited in the patents due to the basic and generic nature of the research, rendering specific apportioning to olaparib impossible. However, the results are a useful indication of the extent of governmental and charitable funding for early pre-development work.

Once included, the funding sources—where these were reported in the publications (usually as part of the acknowledgements or financial disclosures part of articles)—were extracted from primary research publications and details of the funding amounts were searched for in the online pages/databases of the corresponding funders. Currencies were converted into a common currency (GBP £) using 12 month average currency rates to end of year December 2021 [11].

The third method used to identify relevant scientific work was through an FOI request of a public institution cited as being the key research institute involved in the development of olaparib. The ICR responded to the news that the US Food and Drug Administration had approved Olaparib for the treatment of women with BReast CAncer (BRCA)-mutant advanced breast cancer with a statement that its scientific work had underpinned the development of PARP inhibitors [12]. The ICR is a charity and a member institution of the University of London. We submitted a FOI request to ICR (dated 2^nd^ August 2021) to obtain administrative data on the public and philanthropic sources of funding they had received related to the scientific development of the product.

## Results

### Results of the bibliographic searches

**Fig. 1:**
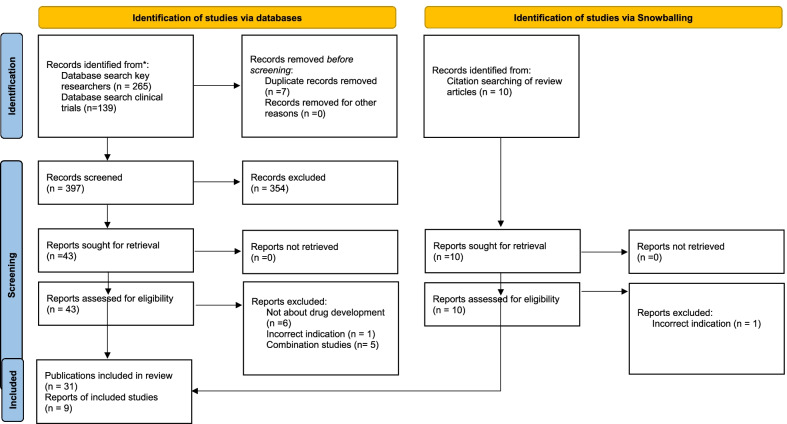
PRISMA flow diagram for bibliographic database searches

The PubMed search on investigator names returned 265 hits; the PubMed product search returned 56 hits. The PubMed searches identified nine review articles [13–21], which helped us in identifying scientific work conducted as part of the development story. On the basis of these review articles, a further ten publications were identified, eight of which were included. Results are shown in Fig. 1.

#### Preclinical research

##### Description

In 2005, two separate groups of researchers demonstrated the potential of PARP1 inhibition as a targeted, synthetic lethal approach to treating BRCA-mutant tumours: the mouse models of Bryant et al. and Farmer et al. [16]. A further preclinical study, McCabe et al. [24], was cited alongside the Farmer work and the Lord team [22]. Three pre-clinical studies were identified that explored BRAC1 and BRAC2 associated breast cancers: Moynahan et al. [26], Tutt et al. [29] and Zhang et al. [17]. A further three pre-clinical studies considered in-vitro and in-vivo synergies: Menear et al. [25], Rottenberg et al. [27] and Takahashi et al. [17]. In total, 10 pivotal studies relating to preclinical work were identified [10, 22–30].

##### Funding sources

Six of these preclinical studies received funding contributions from *charitable organisations*. Five studies named the charities as Cancer Research UK [10, 22–24, 29]; four of these also named Breakthrough Breast Cancer as funders [10, 22, 24, 29]. Farmer, Tutt, McCabe also named the Mary-Jean Mitchell Green Foundation. The charities Yorkshire Cancer Research (Bryant), Swedish Cancer Society (Bryant) and the Dutch Cancer Society (Rottenberg) also contributed to the funding of preclinical research. It was unfortunately not possible to identify specific funding amounts from these charitable organisations based on available information in the public domain.

Four preclinical researchers received public funding from *governmental bodies*—three from the US and one from Europe. Funding from the US Army (DAMD17-98-1-8334) and NIH (CA68425) was received by Moynahan (combined value $2,306,663) [26]. The preclinical research published by Takahashi was supported, in whole or in part, by NIH grants CA72851 (not located) and CA129286 (value of $1,534,769 over 5 years). The work by Takahashi was also supported by the Baylor Scott and White Research Institute, which is the research arm of a non-for-profit healthcare system in Texas [28]. A NIH grant (CA107640) was referenced by Zhang, which between 2004 and 2008 totalled $1,666,357. Harvard Breast Cancer Specialized Program of Research Excellence also contributed an undetermined amount to the preclinical work of Zhang [30]. European public funding from national governmental bodies was cited by Rottenberg [27].

Public funding from the European Union (FP6 programme), Netherlands Organization for Scientific Research, Swiss National Science Foundation and Swiss Foundation for Grants in Biology and Medicine were all named as contributing to the funding of preclinical research. We were able to quantify the EU funding: EU FP6 project CHEMORES (037665) ran from 2007 to 2012 and received an EU contribution of €8,707,358.

Only one publication relating to preclinical research (Menear) did not cite a public funding element to their work [25].

#### Clinical trials

##### Description

We identified one *proof of concept trial* ([31], NCT00494234) and five *phase I studies* ([32], not registered; [33], NCT00777582; [34], NCT00572364; [35], NCT01813474; [36], NCT00516373). We identified six *phase II trials* [37]*:* NCT00494442; [38] NCT00679783; [39] NCT00628251; [40] NCT01078662; [41] NCT00753545; [42] NCT02681562. One *phase III trial* was identified: [43] NCT02000622 (the OlympiAD trial). In addition, we identified 13 publications relating to combination studies, which we have not included as part of product development in this analysis.

##### Funding sources

Three clinical trials cited a combination of industry, government and charity funding for their studies [36, 39, 43]. The government funding was from the UK via the Department of Health/National Institute for Health Research and from the US NIH, whilst charity funding came from Cancer Research UK, Breakthrough Breast Cancer and the Breast Cancer Research Foundation. We were only able to determine a value for the NIH grant, which was a Cancer Center Support Grant CA008748 (representing long-term funding of the research centre), totalling around $25 million per year on an ongoing basis.

Nine clinical trials [31–35, 37, 38, 40, 41] reported that they were solely industry financed, whilst one [42] declared no funding support at all.

### Results of research cited in patents

A web-based search for patents relating to olaparib, using the search term “olaparib”, identified one relevant patent from Europe (EP 2 305 221 B1) and three from the US (US 8,071,579 B2, US 7,151,102 and US 7,449,464). A further seven US patents were identified, which were continuations of previous patents and excluded on this basis.

The European patent EP 2 305 221 B1 referred to 36 scientific citations in support of the application. 23 of the cited scientific works reported receiving public funding, either from government platforms/agencies of individual nations or charities [44–66]. 18 of these publications named the source of public funding but either no specific grant details were provided, or these could not be traced. This included four unspecified NIH grants and one unspecified EU award. One publication referred to an EU-funded project (FIGH-CT-1999–00010), which received an EU public funding contribution of €2,000,000 between 2000 and 2003. The latter publication and an additional four publications declared NIH funding grants (CA094060, CA055914, CA063705, CA074415, GM058986 and GM037706) awarded between 1993 and 2018, with a combined value of $ 60,815,427.

After de-duplicating references common to both patents, as well as those already identified as pre-clinical or clinical research, the US patent US 8,071,579 B2 cited a further 33 scientific publications and 19 of these stated that they had received some form of public funding [67–85]. For 12 of the 19 publications, the name of the funding source was stated but either no specific grant details were provided, or these could not be traced. Among the remaining seven publications, 11 NIH grants were identified, two of which were centre grants (CA023074 and ES006694) and for two, no funding amounts could be found (CM04700 and N01CO5600). Seven grants (CA072008, CA065579, CA043894, CA06294, CA068228, GM60915 and CA084407) were project grants awarded between 1993 and 2018, with a combined value of $79,519,938. In addition, three EU grants were declared (European Cost Action D20/003/00, EU BIOMED2 BMH4-CT-98-3784 and EU RISC-RAD D16R-CT-2003-508843). We were able to trace a grant amount of €10,000,000 awarded between 2004 and 2008 using the EU RISC-RAD D16R-CT-2003-508843 grant identifier (ID); no amounts could be determined for the other two EU grants.

Through the other two US patents (7, 449,464 and 7,151,102) a further 120 references (after removal of duplicates) were identified. 43 of these references could be retrieved and 29 of them referred to public or philanthropic funding. However, in only seven cases could exact public funding amounts be traced. These seven studies referred to six grants (R01HL59266, R21HL065145, GM18640, CA58183, CA30195, CA43318) totalling $3,360,990. These seven studies also included a Swiss Bridge Award (CHF 275,000), a Grants-in-Aid for Cancer Research from the Ministry of Education, Science and Culture in Japan (Japanese ¥ 117,000,000) and an EU 5^th^ Framework project funding of €1,354,856.

Table 1 summarises the number of studies that reported having received public or philanthropic funding, classified by stage of R&D, together with specific values of the funding, where we were able to trace these. The middle column shows the number of studies that reported a public or philanthropic funding contribution and where there was sufficient information available from the funder to quantify this contribution. There is no overlap between the funding amounts associated with different stages of R&D as we accounted for duplicates.

**Table 1:** Public funding for olaparib, by stage of R&D and availability of funding information

	Number of studies receiving public funding (as a % of all studies)	Number of studies where public contribution was quantifiable (as a % of all publicly funded studies)	Value of public project funding that could be identified
Research named in patents	71/112 (63%)	16/71 (23%)	$143,696,353.00 + €13,354,856.00 + Jap Yen ¥117,000,000.00
Pre-clinical research	9/10 (90%)	4/9 (44%)	$5,507,789.00 + €8,707,358.00
Clinical trials (phases I to III)	3/13 (23%)	0/3 (0%)	- (only a center funding grant of $25 million p.a identified through 1 publication.)
Total in common GBP £ currency ($ and €)			£ 128,122,052.50 ($ 176,500,967.70 or € 148,530,086.30)

### Results of research funding to ICR for the development of PARP inhibitors for women with breast cancer

A request for information (dated 2^nd^ August 2021) was sent to the ICR, concerning funding awards relating to the development of PARP inhibitors for women with BRCA-mutant breast cancer. Information kindly supplied by ICR in answering the FOI request is shown in Table 2. Total public funding of this one public sector research institute’s work on PARP inhibitors—that led to the development of olaparib—amounted to just over £400 million. We did not have enough information on the ICR funding grants to identify how much overlap there was between these grants and those listed in the scientific publications.

**Table 2:** Public funding of ICR’s development work on olaparib

Funder’s Name	Funder’s Status	Number of grants	Total sum of grants (£)	Research awards in other currencies
Cancer Research UK	Charity	18	£ 72,861,124.08	
Breast Cancer Now	Charity	6	£ 130,173,644.00	
National Institute for Health Research	National government	4	£ 194,492,365.00	
Prostate Cancer UK	Charity	3	£ 6,874,700.88	
Medical Research Council	National government	2	£ 484,306.12	
Stand Up to Cancer	Charity	2	£ 10,667,84.00	$10,000,000.00
European Commission	Supranational	1	£ 197,754.00	
Prostate Cancer Foundation	Charity	1		$225,000.00
National Health and Medical Research Council of Australia	National government	1	£ 841,075.00	
Sum in original currency		38	£ 406,991,753.00	$10,225,000.00
Total in common GBP £ currency ($ and €)			£ 414,414,081.00 ($ 570,896,929.00 or € 480,424,392.05)	

## Discussion

Olaparib received approval as a monotherapy for the treatment of adult patients with germline *BRCA*1/2-mutations, and who have human epidermal growth factor receptor 2 (HER2)-negative locally advanced or metastatic breast cancer. Treating BRCA-mutated metastatic breast cancer patients has been estimated to cost € 3,794.00 for a 21-day cycle and € 75,889.40 for a course of treatment lasting for a median of 14.5 months (as reported in the OlympiAD trial) [86]. Equivalent price for a 14.5-month course in the UK is £ 73,008.15 [87].

Over £400 million of public and philanthropic funding was received by the public sector research institute that conducted work leading to the development of PARP inhibitors (equivalent to around $570 million or € 480 million). The public and philanthropic funding of basic, preclinical and clinical research, that was reported in publications and was able to be traced through information provided by funders, amounted to a further £ 128 million (equivalent to around $ 177 million or € 149 million).

The range of public and philanthropic R&D sponsored costs we have identified here lies within the range of estimated development costs, of between $43.4 Million and $4.2 billion [6]—especially when one considers that these estimates also include costs of unsuccessful drugs (whereas we have here looked only at one successful drug).

Methodologically, this analysis has shown that the amount of public and philanthropic funding for scientific development reported by a public sector organisation dwarfs any estimates that can be determined from published papers (our previous estimates of public funding based on available bibliographic information was only able to quantify public funding amounts of between €21 and €30 million).

We think the extent of funding of the ICR for later stage drug discovery research, rather than the basic and translational science research that public sector research is usually credited with financing, reveals that later-stage research is funded to a considerable extent by governmental and charitable contributions. This information helps fill a research gap regarding information on the funding of product development organisations in the public sector [6].

This piece of work follows a previous example, where FOI requests have been used to obtain information from academic research institutes [88]. The information returned from the ICR has been invaluable in documenting the extent of charitable contributions—accounting for around half of the public funding amount they received. Little has been documented about the role of charitable funding, so this is an important finding. The ICR funding results are in line with earlier work by Nayak et al. [3] who investigated downstream public sector support by examining patent and drug development histories [3]. They found that about two-fifths of new biologic drugs approved by the FDA between 2008 and 2017 had received financial support from public sector institutions or their spin-offs for late-stage development.

Although generating an exact monetary sum for public and philanthropic funding as a whole is not possible with bibliographic methods (although it may be for specific funders, e.g., NIH, as [89] have shown), this method is able to describe the depth and breadth of public and philanthropic funding from different sources that may have contributed to product development. Depending on the stage of research, we could only determine exact funding amounts for—at most—under half of studies receiving public or philanthropic funding. To enable the tracing of exact public funding contributions, we recommend that all funders—including charities—provide databases of funding information. This should contain, at a minimum, the year of the award, award amount and lead researchers’ names.

This research follows the bibliographic approach to estimating public and philanthropic funding contributions. An alternative framework approach has recently been suggested by Darrow & Light (2021) for assessing the total societal costs of pharmaceuticals, accounting for indirect funding through tax and benefit concessions [90]. We anticipate that this indirect funding landscape is just as complex, and significant, in Europe.

## Limitations

The clinical trials included in the study resulted only from the bibliographic search and snowballing from identified review papers; no complementary search was conducted using other databases.

It was not possible to identify whether there was any overlap between funding sources cited in the publications and the administrative data on funding from the ICR due to a lack of identifying information.

Even where the publication authors provide exact grant numbers and funder details (which is not always the case), the monetary value of the grant is often not publicly listed by funders—which is a considerable limitation of bibliographic analyses. This situation particularly arises, where charitable organisations are the funders.

A further limitation is in apportioning public or philanthropic funding to specific products or applications, especially in the case of research cited in patents. Olaparib can be applied to different types of cancer. Although we have concentrated on breast cancer, there is of course overlap with scientific work used for other indications or indeed for other product areas—especially in the case of earlier pre-patent and pre-clinical work. Similarly, some of the public and philanthropic funding identified relates to the long-term funding of research centres, where the funding can be used for different purposes—again apportioning to one product/application is not possible.

Finally, it would be interesting to know the percentage that the public and philanthropic funding represents of the total development cost of olaparib. Unfortunately there is no publicly available estimative or a statement of the amount of private funding involved and/or total costs of development.

## Conclusions

Bibliographic methods for estimating the monetary value of total public and philanthropic funding have considerable limitations. This is due to incomplete reporting in publications (e.g. missing grant numbers) but also a lack of published data available from funders on the value of awards. The comparison with administrative information obtained through a FOI request shows that bibliographic methods likely under-estimate the scale of public and philanthropic funding.

We have shown the extent of public and philanthropic funding of an organisation in the public sector that was pivotal in product development work. Charitable funding has not received as much attention in the literature as governmental funding. This paper shows around half of non-industry funding contributions to a public sector research centre with a pivotal role in product development was received from the charitable sector.

## Data Availability

Available on request from corresponding author.

## Abbreviations

BRCA1: Breast Cancer gene 1
BRCA2: Breast Cancer gene 2
EU: European Union
FDA: Food and Drug Administration
FOI: Freedom of information
FP6: Sixth Framework Programme for Research of the European Union
FP7: Seventh Framework Programme for Research of the European Union
HER2: Human epidermal growth factor receptor 2
ID: Identifier
ICR: Institute of Cancer Research
NIH: National Institutes of Health
PARP: Poly-ADP ribose polymerase
R&D: Research and development
UK: United Kingdom
US: United States of America
